# Effects of Glucomannan-Enriched, Aronia Juice-Based Supplement on Cellular Antioxidant Enzymes and Membrane Lipid Status in Subjects with Abdominal Obesity

**DOI:** 10.1155/2014/869250

**Published:** 2014-12-09

**Authors:** Nevena Kardum, Gordana Petrović-Oggiano, Marija Takic, Natalija Glibetić, Manja Zec, Jasmina Debeljak-Martacic, Aleksandra Konić-Ristić

**Affiliations:** Centre of Research Excellence in Nutrition and Metabolism, Institute for Medical Research, University of Belgrade, Drive Subotića 4, 11000 Belgrade, Serbia

## Abstract

The aim of this study was to analyze the effects of a 4-week-long consumption of glucomannan-enriched, aronia juice-based supplement on anthropometric parameters, membrane fatty acid profile, and status of antioxidant enzymes in erythrocytes obtained from postmenopausal women with abdominal obesity. Twenty women aged 45–65 with a mean body mass index (BMI) of 36.1 ± 4.4 kg/m^2^ and waist circumference of 104.8 ± 10.1 cm were enrolled. Participants were instructed to consume 100 mL of supplement per day as part of their regular diet. A significant increase in the content of n-3 (*P* < 0.05) polyunsaturated fatty acids in membrane phospholipids was observed, with a marked increase in the level of docosahexaenoic fatty acid (*P* < 0.05). Accordingly, a decrease in the n-6 and n-3 fatty acids ratio was observed (*P* < 0.05). The observed effects were accompanied with an increase in glutathione peroxidase activity (*P* < 0.05). Values for BMI (*P* < 0.001), waist circumference (*P* < 0.001), and systolic blood pressure (*P* < 0.05) were significantly lower after the intervention. The obtained results indicate a positive impact of tested supplement on cellular oxidative damage, blood pressure, and anthropometric indices of obesity.

## 1. Introduction

Obesity is a serious public health problem related to oxidative stress with or without additional diseases, such as insulin resistance, diabetes, polycystic ovary syndrome, and cardiovascular diseases (CVD) [1–3]. Visceral or upper body obesity has been associated with an increased incidence of cardiovascular events. The mechanism in which obesity and development of CVD are rela ted includes metabolic disorders such as dyslipidemia and hyperinsulinemia, disrupted homeostasis, and inflammation [4]. A direct association of obesity with oxidative stress was rationalised by the infiltration of macrophages into adipose tissue and their overproduction of reactive oxygen species (ROS) and cytokines. Additionally, the expansion of fat mass leads to hypoxia and the consequent apoptosis of adipocytes which serves as a trigger for macrophage infiltration [5]. A disbalance in H^+^ levels related to hypoxia has been reported to inhibit mitochondrial production of energy and lead to an incomplete reduction of oxygen and ROS formation [6]. In the state of abdominal obesity, accumulated fat was characterized with disturbed oxidative status accompanied with an increased expression of NAD(P)H oxidase and decreased activity of antioxidant enzymes [7]. It has been shown that central obesity correlates with a decrease in total antioxidant capacity (TAC) independent of age, smoking status, blood pressure, blood lipid, and glucose levels. An association of TAC with body mass index (BMI) and waist circumference was found to be more pronounced in women than in men [8].

Indicators of lipid peroxidation levels are the most common group of indices used to evaluate the individual's oxidative status [9]. Besides the common measurement of lipid peroxidation products, such as malondialdehyde (MDA) and isoprostanes, a relative concentration of polyunsaturated fatty acids (PUFAs) has been suggested as an indirect marker of membrane lipid peroxidation [10, 11]. Due to high content of unsaturated bonds as the main site of free radicals attack, PUFAs are fatty acids susceptible to oxidative damage. Since biological membranes are the first line of cell contact with extracellular substances, including free radicals [12], it is of special importance to determine their fatty acid profile and status of PUFAs. Recent studies revealed that polyphenol-rich plant extracts protect biological membranes from oxidative damage by scavenging free radicals in the medium. Furthermore, their protective effects were related to the incorporation of polyphenols in the outer hydrophilic part of the membrane phospholipid bilayer [12, 13]. This way polyphenols could ensure the usual physicochemical characteristics of the membrane and provide suitable structure and functions.

Chokeberry (*Aronia melanocarpa*) is found to be a rich source of polyphenols, especially anthocyanins present as different forms of cyanidin-glycosides. Numerous health promoting effects of chokeberry, related to its strong antioxidant activity, have been revealed [14]. Beneficial effects of long-term consumption of chokeberry juice and other aronia products on metabolic parameters including lipid profiles, fasting plasma glucose, and blood pressure levels have been reported [15–17].* In vitro* investigations showed an increase in antioxidant defense and decrease in ROS levels after incubation with chokeberry active compounds [18, 19]. However, data on the effects of chokeberry on oxidative status and antioxidant defense* in vivo* are limited. Still, some studies report the stimulation of erythrocytes antioxidant enzymes as a result of chokeberry consumption in humans [15, 20].

Intake of foods rich in dietary fibers has been recommended in order to promote health and prevent cardiovascular and other chronic diseases development. This is based on their positive impact on weight management and traditional risk factors, such as total and LDL cholesterol [21, 22]. Potential role of dietary fibers in defence against oxidative stress has been rarely studied.

The aim of our study is to investigate the effects of a 4-week-long consumption of glucomannan-enriched, aronia juice-based dietary supplement on anthropometric parameters, membrane fatty acid profile, and status of antioxidant enzymes in erythrocytes obtained from female subjects with abdominal obesity.

## 2. Material and Methods

### 2.1. Subjects and Study Design

The study was approved by the Ethical Committee of the Medical Clinical Center in Zemun (Belgrade, Serbia) and has been undertaken according to the Helsinki Declaration. All subjects gave written informed consent prior to the enrolment. Twenty postmenopausal women aged 45–65 with abdominal obesity, defined according to Adult Treatment Panel III [23], as a waist circumference greater than 88 cm, were enrolled. They were instructed to consume 100 mL of glucomannan-enriched, aronia juice-based supplement daily for 4 weeks as part of their regular diet. Exclusion criteria were the presence of chronic diseases treated with drugs, a diagnosis of diabetes, food allergy, dietary supplementation three months preceding the study, or intolerance to the supplement's ingredients.

During the course of the study subjects were instructed to continue with their regular diet. The dietary pattern, assessed by applying 3-month food frequency questionnaire (FFQ), for most of the subjects was defined as “moderately unhealthy,” as characterized by high intake of red meat, added fat, processed food, and low to medium habitual intake of fruits, vegetables, soy, and fish. All subjects were sedentary (<1 h/wk of physical activity), free of alcohol consumption, and nonsmokers with relatively high energy intake.

Blood samples were obtained at the baseline and at the end of the consumption period, after an overnight fast. Erythrocytes were isolated and stored at −80°C for further analysis of antioxidant enzymes status and fatty acid profile. Routine biochemical parameters were determined immediately after blood sampling. Additionally, anthropometric measurements, including blood pressure level, were performed during both study visits.

### 2.2. Glucomannan-Enriched, Aronia Juice-Based Supplement

The intervention drink was donated from Nutrika d.o.o., Belgrade, Serbia. It was prepared from pure aronia juice enriched with 2 g of stable glucomannan fibers (Luralean, Shimizu, Japan). The total quantity of aronia juice provided to the participants was from the same batch. Participants were instructed to keep the beverage in the refrigerator after opening. The content of total phenolics and anthocyanins was determined in our laboratory by previously described methods [24, 25] and obtained values were 586.7 ± 3.3 mg gallic acid equivalent per 100 mL and 15.3 ± 0.2 mg cyanidin-3-glucoside equivalent per 100 mL, respectively.

### 2.3. Sample Collection and Analysis

Venous blood was collected between 8 and 10 pm after the overnight fast into sample tubes for serum and EDTA evacuated tubes at two time points: baseline and the end of a 4-week-long supplement consumption. Lipid status and glucose levels were determined from serum, on the same day the samples were collected, using clinical chemistry analyzer (Cobas e411, Roche Diagnostics, Basel, Switzerland) and Roche Diagnostics Kits according to the manufacturer's instructions. Samples from EDTA tubes were centrifuged to separate the red blood cells from plasma. Packed erythrocytes were washed out three times with cold isotonic saline, divided into aliquots and stored at −80°C for further analysis. Anthropometric parameters, including systolic (SBP) and diastolic blood pressure (DBP) levels, were determined at the beginning and the end of the study.

### 2.4. Fatty Acid Extraction and Analysis

First, lipids were extracted from 0.5 mL of packed erythrocytes with a mixture of chloroform : isopropanol (7 : 11) according to the method described previously [26]. Phospholipids were then separated from other lipid subclasses on a silica thin-layer chromatography plate using the solvent system of petroleum ether, diethyl ether, and glacial acetic acid (87 : 12 : 1, by volume). Afterwards, direct transesterification of fatty acids was carried out according to the previous procedure with slight modifications [27]. The hexane extracts were evaporated under a stream of nitrogen to complete dryness. The residue was dissolved in 10 *μ*L of hexane and 1 *μ*L was injected into the chromatograph.

Methyl esters of fatty acids were analyzed by gas-liquid chromatography in a Shimadzu chromatograph GC 2014 equipped with a flame ionization detector on a Rtx 2330 column (60 m × 0.25 mmID, film thickness 0.2 *μ*m, Restek, Bellefonte, PA). Adequate separation was obtained over a 50 min period with an initial temperature of 140°C held for 5 minutes. The temperature was then increased to 220°C at a rate of 3°C/min and held in the final temperature for 20 minutes. The identification of fatty acid methyl esters was made by comparing peak retention times with standard mixtures (PUFA-2 and/or 37 FAMEs mix, Supelco, Bellefonte, PA). Finally, the content of fatty acids from 16:0 through 22:6n-3 was expressed as a percent of total fatty acids identified.

The percentage of total saturated fatty acids (SFAs) was calculated as the sum of the percentages of C16:0 and C18:0, while the percentage of monounsaturated fatty acids (MUFAs) was determined from C16:1n-7, C18:1n-9, and C18:1n-7 percentages. The percentage of total PUFAs was calculated from the percentages of the individual polyunsaturated long-chain fatty acids C18:2n-6, C20:3n-6, C20:4n-6, C22:4n-6, C20:5n-3, C22:5n-3, and C22:6n-3. The average degree of fatty acid unsaturation (the unsaturation index) was calculated as the average number of double bonds per fatty acid residue multiplied by 100, as previously suggested [28].

### 2.5. Antioxidant Enzyme Analysis

The activity of superoxide dismutase (SOD) in red blood cells was measured using a commercial kit (Randox-Ransod, Cat no. SD 125, UK). The determination of SOD activity was based on superoxide anion (O_2_^−∙^) production in the xanthine-xanthine oxidase system and its further reaction with 2-(4-iodophenyl)-3-(4-nitrophenol)-phenyltetrazolium chloride (INT) which resulted in forming red formazan dye. SOD from samples catalyzed the dismutation of superoxide anion and led to changes in the absorbance, which were monitored continuously at 505 nm and 37°C for 3 min. The units of SOD activity were calculated from these changes with the use of the standard curve made with known amounts of the purified enzyme. Erythrocyte glutathione peroxidase (GPx) activity was determined according to the previously described method [29] with use of commercial kit (Randox-Ransel, Cat no. RS 505, UK). In the presence of cumene hydroperoxide GPx catalyzed the formation of oxidized glutathione, which was further reduced by glutathione reductase with the consumption of coenzyme NADPH+H^+^. Based on this, the activity of GPx was measured by monitoring the decrease in absorbance due to disappearance of NADPH+H^+^ at 340 nm and 37°C. The activity of catalase (CAT) was measured by the method Aebi described [30]. The determination was based on CAT's ability to degrade hydrogen peroxide (H_2_O_2_) with the formation of water and molecular oxygen. A decrease in H_2_O_2_ concentration was measured spectrophotometrically at 230 nm during 3 minutes and the change of absorbance was used for the determination of CAT activity. The activities of antioxidant enzymes were expressed in U/gHb. Cyanmethemoglobin method with Drabkin's reagent based on spectrophotometry at 540 nm was applied for the assessment of hemoglobin concentration in red blood cells [31].

### 2.6. Statistical Analysis

Data are shown as mean values ± standard deviation (SD) and comparisons were performed by paired samples *t*-test. Prior to comparisons, the normality of variables distribution was tested by Shapiro-Wilk test. Analyses were performed using the SPSS software (ver. 15.0; Chicago, IL) and *P* values ≤ 0.05 indicated statistical significance.

## 3. Results

### 3.1. Characteristics of the Subjects

The general characteristics of the study group are presented in Table 1. Twenty postmenopausal women with a mean age of 53.0 ± 5.4 and an average BMI of 36.1 ± 4.4 kg/m^2^ and waist circumference of 104.8 ± 10.1 cm were included. Values of measured biochemical parameters were not changed, except for HDL cholesterol which was slightly decreased. At the end of the consumption period significant decrease in waist circumference (*P* < 0.001), as an indicator of abdominal obesity, and BMI (*P* < 0.001) was recorded. Furthermore, blood pressure level was also evaluated and a lowering effect was noted with a significant change in the case of SBP (*P* < 0.05).

### 3.2. Effects on the Fatty Acid Profile

A significant decrease (*P* < 0.05) was shown in the case of MUFAs, the C18:1n-9, and n-6/n-3 ratio, while an increase was observed in the case of C22:6n-3 (*P* < 0.05), n-3 PUFAs (*P* < 0.05), and unsaturation index (*P* = 0.05). Also, the percentage of total PUFA was lower at the end of the study compared to the baseline, but this difference did not reach statistical significance (*P* = 0.062). Similarly, an insignificant decrease in the amount of total SFA and C16:0 (*P* = 0.066) was noted. These findings are presented in Table 2.

### 3.3. Effects on Antioxidant Enzymes

**Figure 1:**
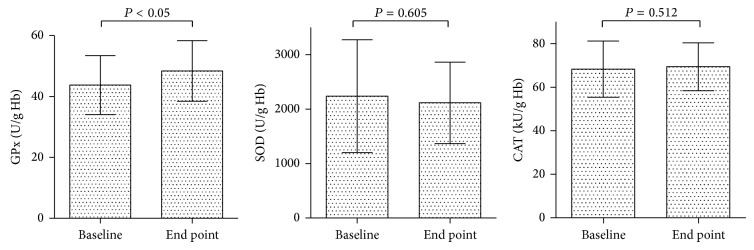
Activities of antioxidant enzymes at the baseline and end point of the study. Data are presented as mean ± SD. GPx = glutathione peroxidase; SOD = superoxide dismutase; CAT = catalase.

Effects of aronia juice-based supplement consumption on antioxidant enzymes in red blood cells are shown in Figure 1. The analysis revealed a statistically significant increase in GPx activity (43.75 ± 9.67 versus 48.39 ± 9.93; *P* = 0.05). However, values of SOD activity did not differ at the end of consumption period compared to the baseline (2237.66 ± 1035.23 versus 2115.15 ± 747.83 U/gHb). Additionally, no impact on CAT activity was recorded (68.28 ± 12.90 versus 69.39 ± 10.99 kU/gHb).

## 4. Discussion

Obesity is associated with increased inflammation, elevated blood lipids and glucose levels, and insensitivity to insulin, which contribute to the initiation and propagation of cardiovascular and other chronic diseases. This is more pronounced in the case of low antioxidant defense and poor antioxidant intake. For these reasons, it would be of special importance to develop interventional strategies for decreasing oxidative stress and incidence of diseases in overweight and obese subjects [2].

In the present study, we investigated the effects of 4-week-long glucomannan-enriched, aronia juice-based supplementation on erythrocytes fatty acids profile in subjects with abdominal obesity. As a result, an increase in the levels of total (*P* = 0.062) and n-3 (*P* < 0.05) PUFAs, followed with an increase in unsaturation index (*P* = 0.05), was observed. An increase in total n-3 PUFAs was most probably based on the increase in relative amount of docosahexaenoic (DHA; C22:6n-3) fatty acid (*P* < 0.05). PUFAs are considered to be the most prone compounds to oxidation with their double bonds as the sites of interaction with free radicals. Our findings demonstrate oxidative damage attenuation manifested in an increase of average unsaturation of fatty acids in the cell membrane. Additionally, the observed results suggest potential beneficial effects of aronia juice-based supplement consumption in the prevention of cardiovascular and other obesity associated diseases. It has been shown that the amount of n-3 fatty acids in erythrocyte membranes is negatively associated with cardiovascular disease risk, incidence of rheumatoid arthritis, and metabolic syndrome [32, 33]. DHA, as the most important individual n-3 fatty acid, showed the potential to lower blood pressure, through the increase in endothelium release of nitric oxide [34]. Following the increase in n-3 fatty acids content, a significant decrease (*P* < 0.05) in relative n-6/n-3 ratio was observed at the end of the consumption period. A high n-6/n-3 ratio has been associated with inflammation and pathogenesis of chronic diseases, while its reducing resulted in attenuation of diseases development [35]. The levels of oleic acid (C18:1n-9) and total MUFAs in erythrocyte phospholipids were significantly decreased after the supplementation (*P* < 0.05). It is possible that MUFAs proportion, along with no significant decrease in SFA content, was lowered as a compensation to the increase of PUFAs.

Several studies have evaluated the physicochemical characteristics of erythrocyte membranes depending on BMI and revealed a statistically significant lower content of n-3 fatty acids and higher n-3/n-6 ratio in obese women compared to those with a normal BMI. Erythrocyte membranes were found to be particularly susceptible to peroxidation in obese subjects, which is accompanied with a decrease in the amount of PUFAs and altered fluidity and structure of membrane [36, 37]. This way, deformed red blood cells exert lower diffusion capacity, which could lead to insufficient tissue oxygenation. Hypoxia of cardiovascular and endothelial cells in particular further contributes to an increasing incidence of all cardiovascular pathologies associated with obesity [36].

The observed effects on fatty acid profiles of membrane phospholipids were accompanied with a significant increase of GPx activity in erythrocytes (*P* < 0.05) at the end of intervention period compared to baseline. No significant changes were noted in the activities of the other two evaluated antioxidant enzymes, SOD and CAT. Our findings were in accordance with the improvement in GPx status, shown in patients with hypercholesterolemia supplemented with aronia anthocyanins for 1 month [38]. Broncel et al. [15] found a significant increase in both SOD and GPx activities after a 2-month-long consumption of chokeberry extract in subjects with metabolic syndrome. Additionally, they reported a significant decrease in the activity of CAT.

Detected increase of average degree of membrane fatty acid unsaturation indicates protective effects of glucomannan-enriched, aronia juice-based supplement on oxidative damage of membrane lipids. In our previous work, we found a significant correlation between increase in GP_X_ activity and relative amount of total PUFAs in erythrocytes membranes of healthy female volunteers who consumed polyphenol-rich aronia juice for 12 weeks [39]. Based on these findings and results obtained from presented study, we suggest indirect antioxidant effects of aronia juice as potential mechanism for observed alterations in membrane fatty acid composition. This could be explained with presence of polyphenols as main bioactive compounds in aronia juice. Indirect effects of polyphenols against oxidative stress were previously suggested, based on their ability to stimulate activity of antioxidant enzymes and modulate cell signaling pathways and gene expression [40]. Additionally, protective effects of polyphenols against membrane lipid oxidation were associated with their interaction with the lipid phase of erythrocyte membrane and incorporation in outer hydrophilic part [12, 13]. As far as authors are aware data on direct effects of glucomannan intake on oxidative status or membrane fatty acid profile in humans are lacking. Limited data include the effects of glucomannan to decrease ROS generation in human neutrophils* in vitro* [41]. Thus, we hypothesize that the antioxidant effects of tested supplement are mainly the result of aronia juice polyphenols action. However, putative antioxidant effects of glucomannan could be mediated through effects on postprandial load of glucose and lipids shown previously [42] and subsequent raise of oxidative stress accompanied with the similar effects of polyphenols [43].

Consumption of glucomannan-enriched, aronia juice-based supplement exerted effects on anthropometric parameters as well. A significant decrease in BMI (*P* < 0.001) and waist circumference (*P* < 0.001) was shown at the end of the intervention period compared to baseline. Relying on the literature data, observed beneficial effects on weight management could be attributed to both aronia juice and glucomannan. After reviewing the data from available human intervention studies, EFSA Panel on Dietetic Products, Nutrition, and Allergies revealed that glucomannan could have effects on body weight loss in the context of an energy-restricted diet [42]. This was explained as a reduced appetite achieved when glucomannan is consumed as a preload before meals. According to EFSA, in order to obtain claimed effects, at least 3 g of glucomannan per day should be administered. However, the reported effects of glucomannan consumption on weight management are not consistent. A recently conducted meta-analysis of 8 randomized clinical trials revealed no statistically significant effects of glucomannan on weight loss in comparison to a placebo [44]. On the other hand, data on the possible effects of chokeberry, and its products, on weight loss in human intervention studies are limited. Some authors suggest that polyphenol-rich foods could contribute to the management of weight control due to their beneficial effects on insulin sensitivity, adipogenesis, and inflammatory pathways. Accordingly, they reported suppressive effects of aronia extracts on weight gain in rats fed for 6 weeks with a high fructose diet with or without the extract added [45].

Four-week-long consumption of glucomannan-enriched, aronia juice-based supplement exerted lowering effects on blood pressure, with a significant decrease observed in SBP levels (*P* < 0.05). Observed hypotensive effects could be associated with weight reduction, since obesity is considered to be risk factor for hypertension and CVD development. Additionally, previous studies reported beneficial effects of aronia products on blood pressure level. Both SBP and DBP were significantly lowered in subjects with metabolic syndrome who consumed aronia extract regularly for two months [15]. Similarly, a decrease in blood pressure level, which was significant for DBP, was observed after a 6-week-long consumption of chokeberry juice in men with mild hypercholesterolemia [17]. Recent studies reported vasorelaxing properties of polyphenols which are most likely based on the stimulation of nitric oxide synthesis in vascular endothelium [46, 47]. In addition, blood pressure lowering effects of aronia products were considered to be an outcome of other activities as well, including the inhibition of angiotensin I-converting enzyme and antioxidative effects [48].

The role of obesity in the pathogenesis of cardiovascular and other chronic diseases relies mostly on its association with other risk factors, including elevated blood glucose and lipid levels, inflammation, and insulin resistance. Deleterious role of obesity on human health is even more pronounced in case of low antioxidant capacity and low intake of dietary antioxidants [2, 4]. Results obtained in this study indicate a positive impact of glucomannan-enriched, aronia juice-based supplement consumption on cellular redox status of subjects with abdominal obesity, shown by the stimulation of GPx activity and alteration in membrane fatty acids. Beneficial effects also include an improvement in anthropometric parameters and blood pressure. Thus, the overall effects of a tested product containing both dietary fibers and antioxidants-rich aronia juice are putatively a result of their synergistic effects on several indices of obesity, and related risk factors. Accordingly it could be suggested as a supplement to the optimal diet in obese subjects, as an effective dietary measure in the prevention of cardiovascular and other chronic diseases related to obesity. Although final conclusions are limited due to the low number of study participants and the lack of proper control this study shows a potential of a novel product and favors its further research.

## Figures and Tables

**Table 1:** Anthropometric and biochemical parameters.

Parameter	Baseline	End point	*P* value
Number	20		
Age (years)	53.0 ± 5.4		
Body weight (kg)	96.1 ± 12.3	93.9 ± 10.7	0.075
BMI (kg/m^2^)	36.1 ± 4.4	35.0 ± 4.0	<0.001
Waist circumference (cm)	104.8 ± 10.1	100.6 ± 9.2	<0.001
SBP (mm Hg)	127.6 ± 16.9	116.4 ± 16.1	<0.05
DBP (mm Hg)	83.5 ± 9.9	78.4 ± 9.1	0.076
Total cholesterol (mmol/L)	6.28 ± 1.41	5.92 ± 1.06	0.164
HDL cholesterol (mmol/L)	1.26 ± 0.32	1.18 ± 0.33	<0.05
LDL cholesterol (mmol/L)	4.05 ± 1.32	3.80 ± 1.0	0.164
Triglycerides (mmol/L)	2.05 ± 0.89	2.05 ± 1.23	0.717
Glucose (mmol/L)	6.45 ± 2.01	6.0 ± 1.38	0.137

Data are presented as mean ± SD.

BMI: body mass index; SBP: systolic blood pressure; DBP: diastolic blood pressure.

**Table 2:** Membrane fatty acid profile in erythrocytes.

Fatty acid (%)	Baseline	End point	*P* value
Saturated	42.33 ± 5.20	40.58 ± 3.51	0.125
16:0	22.22 ± 4.54	20.24 ± 2.45	0.066
18:0	19.96 ± 2.61	20.34 ± 2.53	0.655
Monounsaturated	15.89 ± 2.10	14.79 ± 1.55	<0.05
16:1n-7	0.31 ± 0.16	0.34 ± 0.14	0.483
18:1n-9	13.99 ± 1.94	12.94 ± 1.44	<0.05
18:1n-7	1.58 ± 0.42	1.51 ± 0.24	0.414
n-6 polyunsaturated	35.86 ± 5.72	37.58 ± 3.16	0.108
18:2n-6	12.50 ± 1.35	12.34 ± 1.67	0.591
20:3n-6	2.20 ± 0.57	2.09 ± 0.56	0.557
20:4n-6	17.13 ± 4.27	18.28 ± 2.49	0.090
22:4n-6	4.48 ± 1.26	4.74 ± 0.83	0.273
n-3 polyunsaturated	5.91 ± 1.59	7.05 ± 2.16	<0.05
20:5n-3	0.32 ± 0.19	0.44 ± 0.47	0.171
22:5n-3	1.71 ± 0.41	1.87 ± 0.26	0.227
22:6n-3	3.89 ± 1.24	4.74 ± 1.60	<0.05
Total polyunsaturated	41.77 ± 6.69	44.63 ± 3.89	0.062
n-6/n-3	6.51 ± 1.96	5.72 ± 1.48	<0.05
Unsaturation index	169.62 ± 25.42	178.81 ± 16.60	0.05

Data are presented as mean ± SD.
